# Trend analysis of palliative care consultation service for terminally ill non-cancer patients in Taiwan: a 9-year observational study

**DOI:** 10.1186/s12904-021-00879-z

**Published:** 2021-11-25

**Authors:** Lian-Shin Lin, Ling-Hui Huang, Yu-Chen Chang, Chun-Li Wang, Lung-Chun Lee, Chung-Chieh Hu, Pi-Shan Hsu, Wei-Min Chu

**Affiliations:** 1 Department of Nursing, Taichung Veterans General Hospital, Taichung, Taiwan; 2 Technology Transfer and Incubation Center, National Health Research Institutes, Miaoli, Taiwan; 3 Department of Family Medicine, Taichung Veterans General Hospital, Taichung, Taiwan; 4 Institute of Medicine, Chung Shan Medical University, Taichung, Taiwan; 5 Department of Occupational Medicine, Taichung Veterans General Hospital, Taichung, Taiwan; 6 School of Medicine, National Yang Ming Chiao Tung University, Taipei, Taiwan; 7 School of Medicine, Chung Shan Medical University, Taichung, Taiwan; 8 Institue of Health Policy and Management, National Taiwan University, Taipei, Taiwan

**Keywords:** Palliative care consultation service, Non-cancer, Do-not-resuscitate, Awareness

## Abstract

**Backgrounds:**

Early integration of palliative care for terminally ill non-cancer patients improves quality of life. However, there are scanty data on Palliative Care Consultation Service (PCCS) among non-cancer patients.

**Methods:**

In this 9-year observational study Data were collected from the Hospice-Palliative Clinical Database (HPCD) of Taichung Veterans General Hospital (TCVGH). Terminally ill non-cancer patients with 9 categories of diagnoses who received PCCS during 2011 to 2019 were enrolled. Trend analysis was performed to evaluate differences in categories of diagnosis throughout study period, duration of PCCS, patient outcomes, DNR declaration, awareness of disease by patients and families before and after PCCS.

**Results:**

In total, 536 non-cancer patients received PCCS from 2011 to 2019 with an average age of 70.7 years. The average duration of PCCS was 18.4 days. The distributions of age, gender, patient outcomes, family’s awareness of disease before PCCS, and patient’s awareness of disease after PCCS were significantly different among the diagnoses. Organic brain disease and Chronic kidney disease (CKD) were the most prevalent diagnoses in patients receiving PCCS in 2019. For DNR declaration, the percentage of patients signing DNR before PCCS remained high throughout the study period (92.8% in 2019). Patient outcomes varied according to the disease diagnoses.

**Conclusion:**

This 9-year observational study showed that the trend of PCCS among non-cancer patients had changed over the duration of the study. An increasing number of terminally ill non-cancer patients received PCCS during late life, thereby increasing the awareness of disease for both patients and families, which would tend to better prepare terminally ill patients for end-of-life as they may consider DNR consent. Early integration of PCCS into ordinary care for terminally non-cancer patients is essential for better quality of life.

**Supplementary Information:**

The online version contains supplementary material available at 10.1186/s12904-021-00879-z.

## Background

The world is rapidly aging [1]. Because of improvements in public health and medical interventions, life expectancy has increased worldwide in recent decades, and continues to rise in many countries [2]. Thus, causes of death from communicable causes to non-communicable diseases are also changing due to increased life expectancy [3]. Cancer accounts for a large proportion of deaths annually, but non-cancer terminal disease also produces disease burden and poor quality of life during late life [4].

Palliative care has been demonstrated to relieve symptoms near end-of-life [5], to overcome psychological distress [6], and to improve quality of life for cancer patients and their family members [7, 8]. In recent years, the importance of palliative care for non-cancer terminally ill patients has been brought to light in multiple studies [9–11]. Studies have shown that palliative care among non-cancer patients can reduce rates of emergency department visits, admissions to hospital, and admissions to the intensive care unit during end-of-life [12]. A previous study also showed that early integration of palliative care for patients with diseases other than cancer can improve breathlessness [13], increase Do-Not-Resuscitate (DNR) consent, and increase patients’ and families’ recognition of the diagnosis [14].

Multiple palliative care services have been initiated in recent years, including inpatient care in palliative care unit [15], palliative home care service [16] and palliative care consultation service (PCCS) [17]. A previous study has shown that PCCS during hospitalization for cancer patients can improve patients’ and families’ awareness of disease diagnosis and prognosis, and also increase consent to DNR [18]. Also, symptom control was better when receiving PCCS among cancer patients [19].

Palliative care service has been implemented in Taiwan’s National Health Insurance (NHI) since 1996, and includes palliative home care (introduced in 1996), palliative inpatient care (2000), and PCCS (2005) [20]. In 2009, the service was expanded by allowing non-cancer terminally ill patients into the PCCS service, which is covered by the NHI program. When non-cancer terminally ill patients are admitted to hospital, they are cared for by a disease specialist as well as a palliative care specialist and a palliative care team, consisting of a nurse, a consulting psychologist, a social worker, and a volunteer. Since 2011, Taiwan has implemented significant environmental changes in palliative care, including the amendments to “Hospice Palliative Care Act” in 2011 and 2013 [21], and further announcement and execution of the “Patient Right to Autonomy Act” in 2016 and 2019 [22].

However, there are few data on PCCS for non-cancer terminally ill patients, especially in Asian countries. Thus, it’s difficult to evaluate the impact of PCCS on the non-cancer population. In this 9-year observational study, we aimed to evaluate the trend and impact of PCCS, including trend of diagnosis of non-cancer terminal disease, duration of PCCS in different disease categories, DNR declaration before and after PCCS, patients’ and families’ awareness of disease before and after PCCS, and patient outcomes among non-cancer terminally-ill patients.

## Methods

### Data sources

Data were collected from the Hospice-Palliative Clinical Database (HPCD) of Taichung Veterans General Hospital (TCVGH). TCVGH is the only public tertiary medical center in central Taiwan with more than 1500 beds in the hospital. The palliative care team in TCVGH was established in 2003, with team members that include physicians, nurses, consulting psychotherapists, social workers, spiritual therapists, art therapists and trained volunteers. The palliative care team provides comprehensive hospice-palliative care in 3 different settings, including inpatient palliative care service, PCCS, and palliative home care service.

### Study group identification

We enrolled terminally ill non-cancer patients who were admitted to TCVGH and referred to PCCS from Jan 2011 to Dec 2019. Since 2009, there were 9 specific diagnosis, including dementia, other brain disease (terminal condition such as severe stroke, severe brain injury, multiple sclerosis, Parkinson’s disease and Huntington’s disease), heart failure, chronic obstructive pulmonary disease (COPD), other diseases of the lung, chronic liver disease or cirrhosis, acute kidney injury, chronic kidney disease and motor neuron disease, which were eligible for PCCS coverage by Taiwan’s NHI. For non-cancer terminally ill patients in need, his/her visiting staff would consult doctor of PCCS team initially. During consultation, the palliative care physician and nurse went to visit the patient and recorded their chief complaints, present illnesses, active problems, and previous experiences of treatment, and initiated talks on the value and preference to the patient and family. Also, after carefully evaluation, suggestion regarding patient’s medication, physical care and mental care would be given by PCCS team members to patient’s original care team.

### Research variables

All data of enrolled patients were collected and extracted from the HPCD, including age at admission, gender, major diagnosis, date of the last admission, date of PCCS enrollment, duration of PCCS service, DNR order status, date of DNR declaration, patients’ and families’ awareness of disease before and after PCCS, patient outcomes, and date of discharge or death.

The data for the duration of PCCS was determined by the interval between the date of first enrollment in PCCS and date of discharge or PCCS termination. DNR order rate (in percent) was calculated as the number of patients who signed a DNR order divided by the number of patients who received PCCS each year. The number of DNR declaration before PCCS and after PCCS was also documented. The awareness of disease was assessed by PCCS nursing staff for each patient and one of their family before and after PCCS.

### Statistical analysis

Statistical analyses were performed using SAS version 9.4 (Statistical Analysis Software 9.4, SAS Institute Inc., Cary, North Carolina, USA). Gender distributions, diagnosis distributions, DNR rates, awareness of disease and patient outcomes were analyzed using chi-square tests. A two-tailed *p* value < 0.05 was considered statistically significant.

## Results

Table 1 shows the trends of all terminally ill non-cancer patients who were admitted to TCVGH and received PCCS during hospitalization. In total, 536 patients were enrolled from 2011 to 2019 with an average age of 70.7 years. The average duration of PCCS was 18.4 days. The percentages of DNR declaration before and after PCCS was 82.8 and 15.1%, respectively. The DNR order status as well as the patient outcomes differed significantly between different years.

**Table 1:** Characteristics of terminally Ill non-cancer patients receiving PCCS from 2011 to 2019

Characteristics	total *n* = 536		2011 *n* = 2		2012 *n* = 3		2013 *n* = 7		2014 *n* = 15		2015 *n* = 81		2016 *n* = 81		2017 *n* = 130		2018 *n* = 120		2019 *n* = 97		*p*
Gender, n (%)																					0.1891
Men	323	(60.26)	2	(100)	1	(33.33)	6	(85.71)	7	(46.67)	51	(62.96)	53	(65.43)	85	(65.38)	67	(55.83)	51	(52.58)	
Women	213	(39.74)	0	(0)	2	(66.67)	1	(14.29)	8	(53.33)	30	(37.04)	28	(34.57)	45	(34.62)	53	(44.17)	46	(47.42)	
Age, mean (sd)	70.71	(19.1)	53.50	(6.36)	69.00	(13.89)	69.86	(16.85)	73.13	(17.81)	68.42	(17.5)	66.65	(23.55)	68.61	(20.3)	72.63	(18.27)	76.56	(14.48)	0.0153
Length of days of PCCS (sd)	18.42	(36.83)	14.00	(8.49)	20.00	(20.3)	5.57	(3.05)	19.87	(15.46)	19.75	(19.17)	16.83	(17.47)	24.95	(68.5)	15.92	(16.77)	13.75	(14.07)	0.3986
DNR declaration, n (%)																					0.0181
Not signed	11	(2.05)	0	(0)	0	(0)	0	(0)	0	(0)	4	(4.94)	3.00	(3.7)	1.00	(0.77)	3.00	(2.5)	0.00	(0)	
Signed before PCCS	444	(82.84)	2	(100)	2	(66.67)	7	(100)	9	(60)	57	(70.37)	68.00	(83.95)	108.00	(83.08)	101.00	(84.17)	90.00	(92.78)	
Signed after PCCS	81	(15.11)	0	(0)	1	(33.33)	0	(0)	6	(40)	20	(24.69)	10.00	(12.35)	21.00	(16.15)	16.00	(13.33)	7.00	(7.22)	
Disease category, n (%)																					0.0133
Heart failure	50	(9.33)	0	(0)	0	(0)	1	(14.29)	2	(13.33)	6	(7.41)	10	(12.35)	11	(8.46)	12	(10)	8	(8.25)	
Other brain disease	159	(29.66)	0	(0)	0	(0)	3	(42.86)	3	(20)	29	(35.8)	23	(28.4)	43	(33.08)	31	(25.83)	27	(27.84)	
Dementia	28	(5.22)	0	(0)	0	(0)	0	(0)	0	(0)	3	(3.7)	3	(3.7)	2	(1.54)	10	(8.33)	10	(10.31)	
Chronic obstructive lung disease	34	(6.34)	0	(0)	0	(0)	0	(0)	2	(13.33)	4	(4.94)	4	(4.94)	9	(6.92)	9	(7.5)	6	(6.19)	
Other disease of lung	73	(13.62)	0	(0)	1	(33.33)	2	(28.57)	1	(6.67)	12	(14.81)	11	(13.58)	24	(18.46)	14	(11.67)	8	(8.25)	
Acute kidney injury	25	(4.66)	0	(0)	0	(0)	1	(14.29)	0	(0)	7	(8.64)	7	(8.64)	5	(3.85)	3	(2.5)	2	(2.06)	
Chronic kidney disease	100	(18.66)	0	(0)	1	(33.33)	0	(0)	4	(26.67)	9	(11.11)	6	(7.41)	22	(16.92)	27	(22.5)	31	(31.96)	
Chronic liver disease and cirrhosis	64	(11.94)	2	(100)	1	(33.33)	0	(0)	3	(20)	11	(13.58)	17	(20.99)	13	(10)	12	(10)	5	(5.15)	
Motor neuron disease	3	(0.56)	0	(0)	0	(0)	0	(0)	0	(0)	0	(0)	0	(0)	1	(0.77)	2	(1.67)	0	(0)	

Supplementary Fig. 1 showed trends of all terminally ill non-cancer patients who received PCCS and not received PCCS. In total, 4153 patients were died of non-cancer diseases of 9 categories. From 2011 to 2019, PCCS rate elevated from 0.36% to 27.71% and the average PCCS rate of non-cancer terminally patients was 12.9%.

Table 2 shows the descriptive data by different categories of non-cancer diseases. The distributions of age, gender, patient outcomes, family’s awareness of disease before PCCS, and patient’s awareness of disease after PCCS were significantly different among the different diagnoses. However, the duration in days for receiving PCCS and DNR order status were not significantly different.

**Table 2:** Characteristics of patients with different disease category receiving PCCS from 2011 to 2019

Characteristics	Heart failure		Other brain disease		Dementia		Chronic obstructive lung disease		Other disease of lung		Acute kidney injury		Chronic kidney disease		Chronic liver disease and cirrhosis		Motor neuron disease		*p*
Gender, n (%)																			<.0001
Men	28	(56)	91	(57.2)	12	(42.9)	32	(94.1)	47	(64.4)	13	(52)	51	(51)	48	(75)	1	(33.3)	
Women	22	(44)	68	(42.8)	16	(57.1)	2	(5.9)	26	(35.6)	12	(48)	49	(49)	16	(25)	2	(66.7)	
Age, mean (sd)	72.08	(17.27)	62.79	(21.65)	85.71	(9.32)	74.91	(15.99)	78.05	(11.52)	71.08	(22.61)	78.40	(13.09)	60.31	(19.15)	64.00	(1)	<.0001
Length of days of PCCS, mean (sd)	17.54	(20.95)	21.23	(23.61)	16.18	(10.2)	17.44	(18.42)	24.88	(87.71)	11.84	(11.82)	14.39	(15.93)	15.25	(13.47)	16.67	(16.77)	0.43
DNR declaration, n (%)																			0.2331
Not signed	2	(4)	2	(1.3)	1	(3.6)	0	(0)	1	(1.4)	1	(4)	1	(1)	3	(4.7)	0	(0)	
Signed																			
Before PCCS	44	(88)	130	(81.8)	23	(82.1)	29	(85.3)	65	(89.0)	16	(64)	87	(87)	47	(73.4)	3	(100)	
After PCCS	4	(8)	27	(16.9)	4	(14.3)	5	(14.7)	7	(9.6)	8	(32)	12	(12)	14	(21.9)	0	(0)	
Patient outcomes																			<.0001
Discharge	5	(10)	18	(11.3)	4	(14.3)	5	(14.7)	6	(8.2)	0	(0)	7	(7)	9	(14)	0	(0)	
Impending Death Discharge	9	(18)	32	(20.1)	1	(3.6)	6	(17.6)	20	(27.4)	9	(36)	15	(15)	22	(34.3)	0	(0)	
Refer to Palliative Home Care Service	7	(14)	4	(2.5)	12	(42.9)	7	(20.6)	5	(6.8)	0	(0)	5	(5)	3	(4.7)	1	(33.3)	
Refer to Palliative Care Unit	6	(12)	15	(9.4)	4	(14.3)	0	(0)	3	(4.1)	2	(8)	22	(22)	8	(12.5)	0	(0)	
Refer to Home Care Service	0	(0)	1	(0.6)	0	(0)	1	(2.9)	0	(0)	0	(0)	2	(2)	0	(0)	0	(0)	
Improved Condition	0	(0)	4	(2.5)	0	(0)	0	(0)	0	(0)	1	(4)	3	(3)	0	(0)	0	(0)	
Death	18	(36)	64	(40.3)	4	(14.3)	11	(32.4)	35	(47.9)	11	(44)	40	(40)	19	(14)	2	(66.7)	
Others	5	(10)	21	(13.2)	3	(10.7)	4	(11.8)	4	(5.5)	2	(8)	6	(6)	3	(4.7)	0	(0)	

Patients’ and families’ awareness of disease are shown in Table 3. Families’ awareness of disease before PCCS and patients’ awareness of disease after PCCS were significantly different in different disease categories. After PCCS, families’ awareness of disease were nearly 100% for all disease categories, however, patients’ awareness of disease after PCCS were still low for some disease categories, especially for dementia (40%) and COPD (43%).

**Table 3:** Awareness of disease among patients and family with different disease category before and after PCCS

Characteristics	Heart failure	Other brain disease	Dementia	Chronic obstructive lung disease	Other disease of lung	Acute kidney injury	Chronic kidney disease	Chronic liver disease and cirrhosis	Motor neuron disease	*p*
Patient’s awareness of disease before PCCS	46%	18%	20%	27%	31%	43%	44%	32%	67%	0.5703
Family’s awareness of disease before PCCS	72%	65%	71%	79%	93%	68%	72%	65%	67%	0.004
Patient’s awareness of disease after PCCS	69%	86%	40%	43%	64%	88%	82%	81%	100%	0.0251
Family’s awareness of disease after PCCS	100%	99%	100%	100%	100%	100%	99%	100%	100%	0.9686

**Fig. 1:**
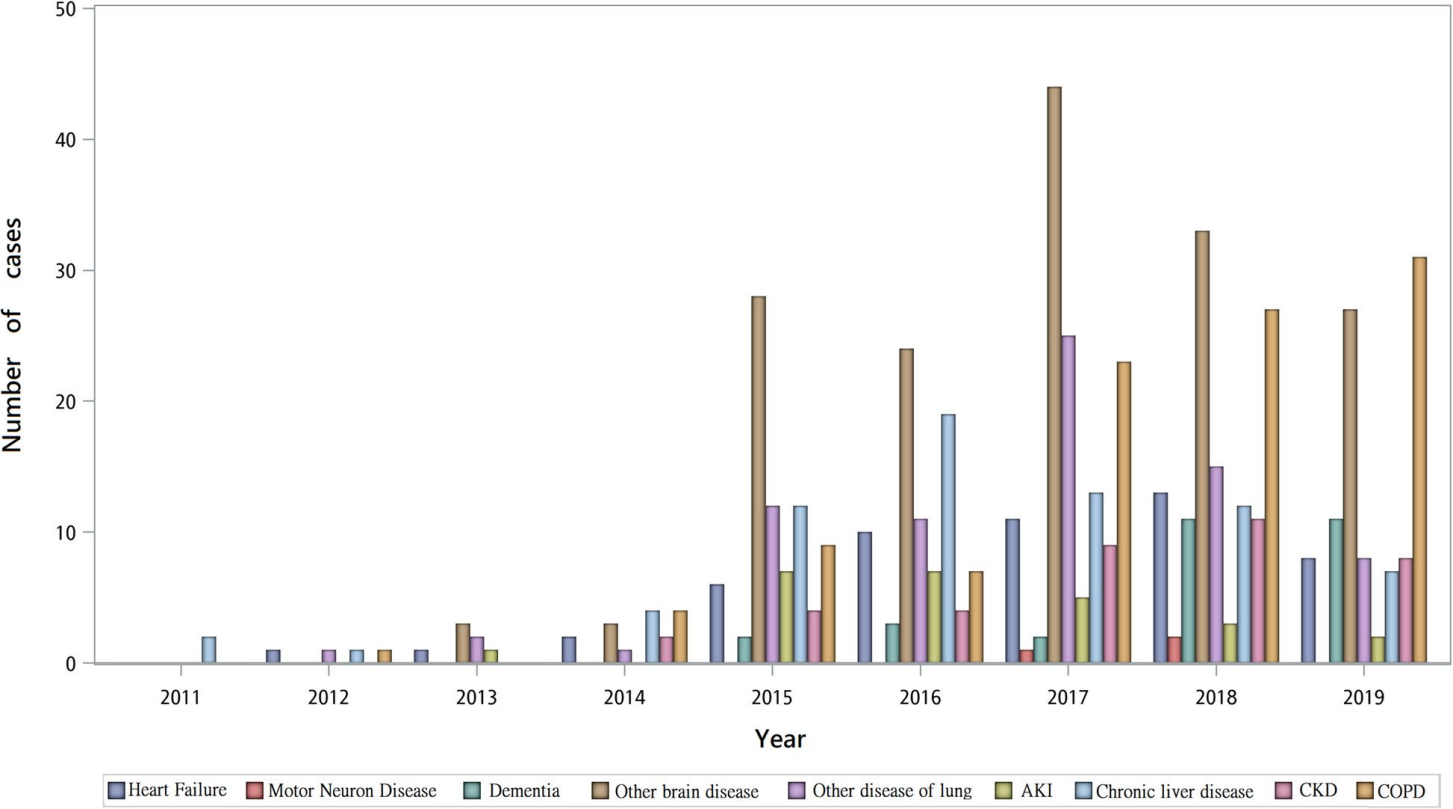
Trend of terminally ill non-cancer patients receiving PCCS with different disease category from 2011 to 2019

**Fig. 2:**
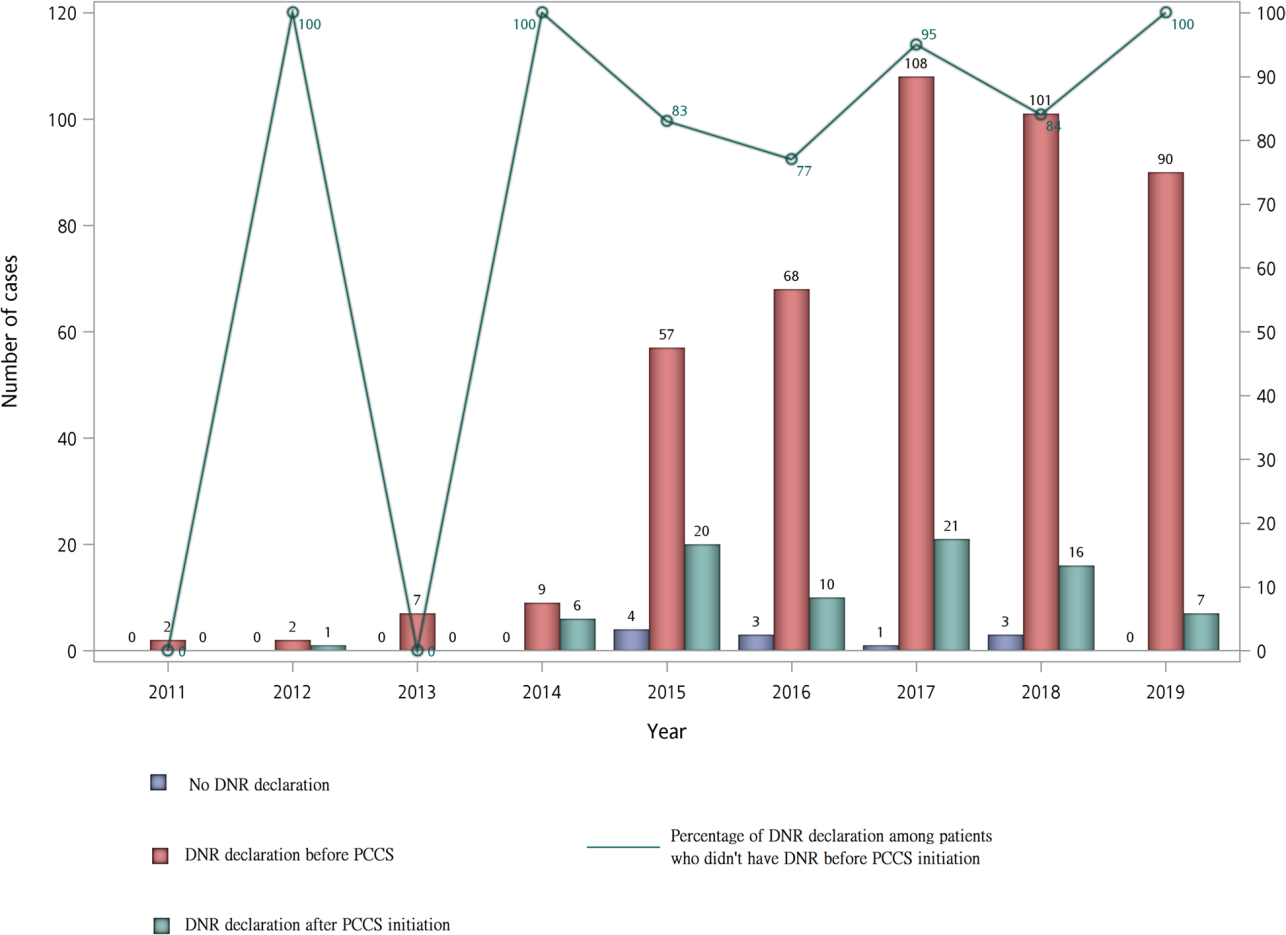
Trend of DNR declaration of terminally ill non-cancer patients receiving PCCS from 2011 to 2019

Figure 1 summarizes the trends of patients in different categories of diseases and receiving PCCS from 2011 to 2019. There was a surge of patients with organic brain disease in 2015, and the number of patients with chronic kidney disease increased gradually from 2015 to 2019. Figure 2 shows the DNR status annually. The percentage of patients signing a DNR before PCCS remained high throughout the duration of the study (92.8% in 2019), but the percentage of those patients who did not sign a DNR before PCCS, but signed after PCCS was also high (100% in 2019).

**Fig. 3:**
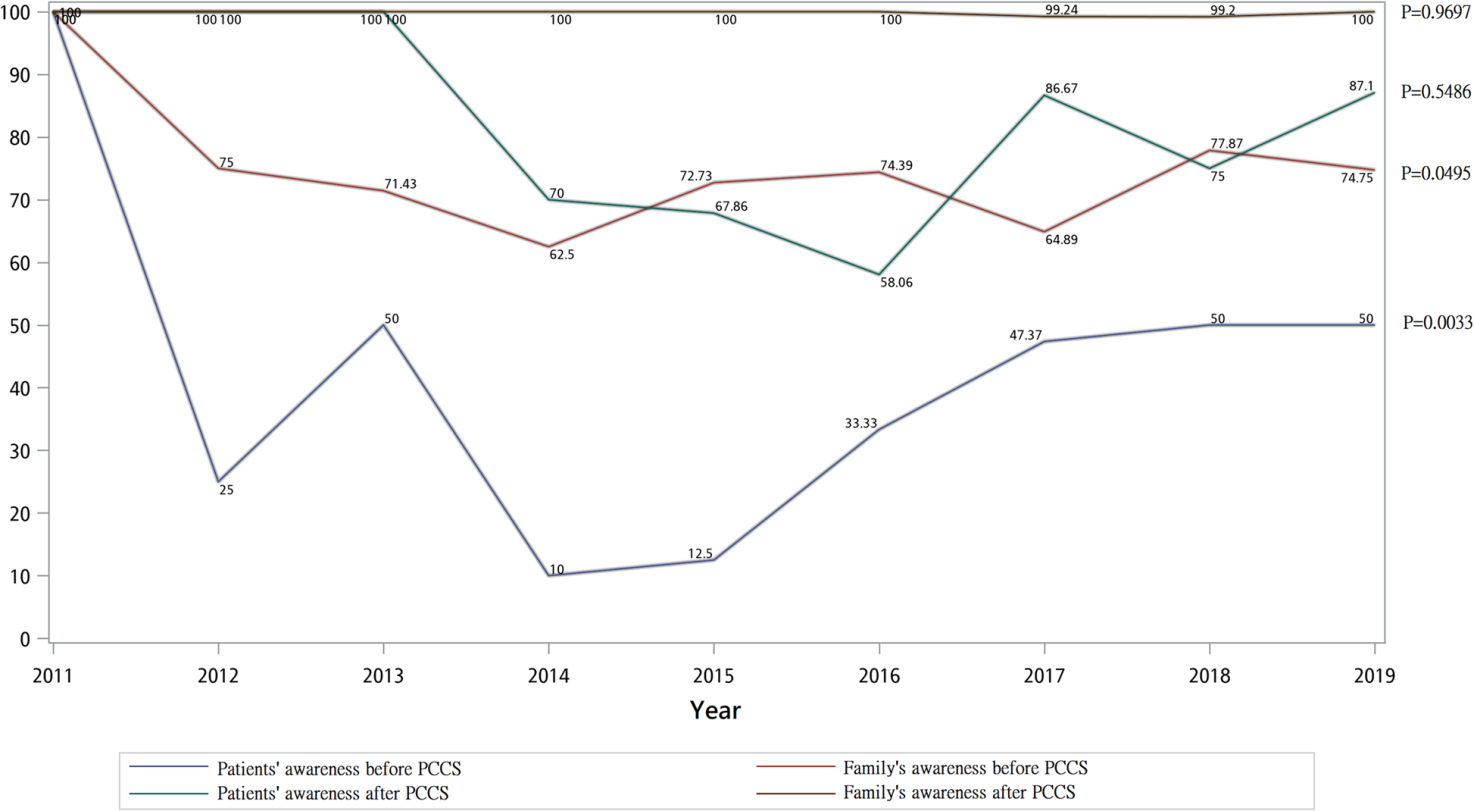
Trend of non-cancer patients’ and family’s awareness of disease before and after PCCS from 2011 to 2019

**Fig. 4:**
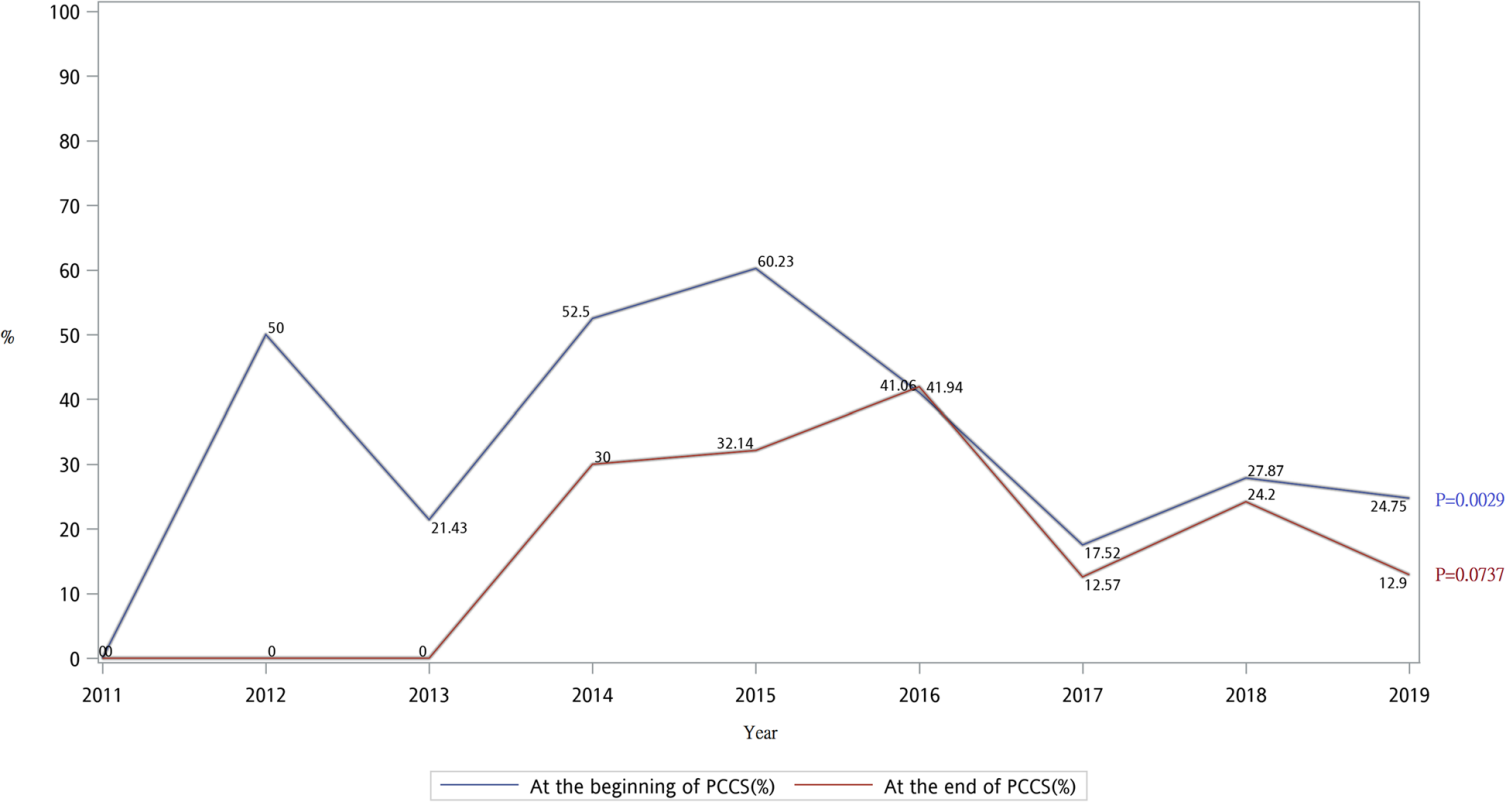
Trend of difference of non-cancer patients’ and family’s awareness of disease before and after PCCS from 2011 to 2019

The awareness of disease among patients and families is shown in Fig. 3. Awareness of disease among family members was consistently higher than that of patients. Both patients’ and families’ awareness of disease significantly increased after PCCS. Figure 4 shows the trend of difference s in awareness of disease between patients and families before and after PCCS. From 2011 to 2019, the difference decreased significantly especially before PCCS.

## Discussion

The main findings of this 9-year observational study are as follows:

1. The number of non-cancer patients receiving PCCS increased during the past decade.
2. The trend of patients’ non-cancer terminal diagnosis in PCCS program changed. Since 2015, organic brain disease was the leading diagnosis of non-cancer patients receiving PCCS. However, chronic kidney disease has increased rapidly since 2017.
3. Patient outcomes differed among the various categories of non-cancer diseases.
4. An increasing number of non-cancer patients signed a DNR consent form before PCCS, but for those who did not sign a DNR consent form, most of them signed after PCCS.
5. PCCS increased disease awareness in both patients and their families. PCCS decreased the difference in disease awareness between patients and families The difference in disease awareness between patients and families before and after PCCS gradually converged.

In our study, the number of non-cancer patients receiving PCCS increased, and rate of receiving PCCS among non-cancer terminally ill patients was also increased. More that, we also found increased service of palliative home care and palliative inpatient care during study period (Supplementary Fig. 2). Among the 3 services of palliative care, the increase of PCCS was the most significant, compare to palliative home care and palliative inpatient care, suggesting that PCCS is a good portal for non-cancer terminally ill patients who have needs for palliative care. This was consistent with a previous study which showed that non-cancer terminal patients’ need for palliative care was increasing. Gadoud et al. observed that palliative care increased among patients with chronic obstructive lung disease and heart failure in the UK from 2009 to 2014 [9]. Hess et al. in Germany found that the proportion of non-cancer patients receiving palliative care increased from 3.5 to 8.1% from 2007 to 2011 [23]. However, both studies pointed out the inadequacy of palliative care service for non-cancer patients. In our study, the average length of PCCS was 18.42 days, suggesting that there is a room for earlier palliative approach. We believe that education and training are key factors for it. In fact, we took an approach of “nurse consultation” for facilitating earlier palliative care in recent years. “Nurse consultation” means that not only physician could initiate the consultation to palliative care team, but also nurses who could do it. Our future study will put emphasis on the effectiveness of this novel approach.

We believe that changes in government policy and the patients’ environment led to rising trend in the number of organic brain disease and chronic kidney disease patients receiving PCCS over time. Taiwan is the first East Asian country to legislate withdrawal of life-sustaining treatment in patients at end-of-life. In 2013, the amendment of the Hospice Palliative Care Act allowed the withdrawal of artificial ventilation in terminally ill patients. Since then, the number of patients with an organic brain lesion who received withdrawal of life-sustaining treatment and palliative care increased [24]. In a previous study in Taiwan, Chang et al. discovered that most patients receiving withdrawal of artificial ventilation were non-cancer patients [25]. As for patients with CKD receiving PCCS, a previous study has shown that among patients with advanced CKD receiving palliative care, there were fewer ICU admissions and cardiopulmonary resuscitation sessions compared with those without palliative care [26]. Also, the “Hospice Palliative Care Act” and “Patient Right to Autonomy Act” helped to establish an environment conductive to better palliative care [27]. In 2016, National Taiwan University Hospital published “Guidelines for hospice palliative care to terminally ill patients with chronic kidney disease at end of life”, which suggested integration of early palliative care for all patients with CKD. From our experiences, we believed that PCCS could also have a teaching and training role in the hospital to train hospital healthcare professionals, like physicians and nurses, to provide generalist palliative care to patients before they are terminally ill. Thus, since 2020, we designed a “Life and death experience workshop” for young doctors and nurses to help them understand how to deal with terminally ill patients and families inter-professionally and how PCCS could benefit.

DNR consent increases quality of life for terminally ill patients [28], and a previous study reported that PCCS could significantly increase DNR consent among terminally ill cancer patients [18]. Our results were consistent with a previous study which demonstrated that PCCS increased DNR designation significantly in patients with cancers or non-cancer diseases [29]. A possible explanation could be that cancer patients’ and families’ awareness of disease and prognosis may have been increased by PCCS [30]. Further study is needed to explore how PCCS improves DNR consideration in non-cancer patients.

We found that there was a significant difference in awareness of disease between patients and their families, and family members possessed higher awareness of disease compared with the patients. This was an interesting finding because we can logically expect cancer patients in Taiwan to have lower disease awareness [18]. The reason is that doctors traditionally tended to inform family members of a cancer diagnosis rather than the patients themselves [31, 32]. However, we also found the same situation in non-cancer patients. To the best of our knowledge, there has been no research exploring the relationship between PCCS and disease awareness among non-cancer patients. It has been reported that PCCS can improve family care burden not only in cancer patients but also in non-cancer patients [33]. Future research is warranted to better understand this issue.

After analyzing patient outcomes in non-cancer patients, we found that patients with chronic kidney disease and organic brain disease had more chances to be referred to palliative care unit, while dementia patients were more referred to palliative home care. We believed that this was due to the relatively long trajectory of diseases for patient with chronic kidney disease and organic brain disease, and thus they have more time to think about quality of care with discussion with care providers [34–36]. For demented patients, palliative home care is needed because recurrent biological and psychosocial symptoms [37]. We also found that patients with organic brain disease and chronic liver disease had more opportunity for impending death discharge, and we thought that this could be related to relatively stable condition with less symptom during end-of-life, and family members could accept care at home during end-of-life period.

This study is the first study to analyze the effect of PCCS and the relationships among awareness of disease and patient outcomes in non-cancer patients. However, there were several limitations in this study. First, the data were collected from a single tertiary center in central Taiwan, so there could have been selection bias of participants and the external validity could therefore be limited. Second, data regarding comorbidities and sociodemographic data were lacking, so we could not analyze the possible effects of comorbidities and sociodemographic condition on awareness of disease, DNR consent, duration of PCCS and patient outcomes.

## Conclusion

This 9-year observational study showed that the trend of PCCS among non-cancer patients changed over the duration of the study. The number of terminally ill non-cancer patients receiving PCCS during late life rose. PCCS increased awareness of disease in both patients and families, and helped to better prepare terminally ill patients for end-of-life by increasing the likelihood of considering DNR consent. We believe that PCCS will become more important for non-cancer patients as societies in developed nations continue to age. Furthermore, the integration of PCCS into ordinary care for terminally ill non-cancer patients is essential for better quality of life.

## Supplementary Information

**Additional file 1: Supplementary Figure 1.** Trend of Terminally Ill Non-Cancer Patients With/Without PCCS from 2011 to 2019. **Supplementary Figure 2.** Trend of Terminally Ill Non-Cancer Patients Receiving PCCS, Palliative Home Care and Palliative Inpatient Care from 2011 to 2019.

## Data Availability

The datasets used and analyzed during the current study are not publicity available, but are available from the corresponding author on reasonable request with the permission of Taichung Veterans General Hospital, Taiwan.

## Abbreviations

DNR: Do-Not-Resuscitate
PCCS: Palliative Care Consultation Service (PCCS)
CKD: Chronic Kidney Disease
HPCD: Hospice-Palliative Clinical Database
TCVGH: Taichung Veterans General Hospital
NHI: National Health Insurance
COPD: Chronic Obstructive Lung Disease
