# Supplementary material for: Trend analysis of palliative care consultation service for terminally ill non-cancer patients in Taiwan: a 9-year observational study
Source: BMC Palliat Care. 2021 Nov 25;20:181. doi: 10.1186/s12904-021-00879-z (PMC8614035; doi:10.1186/s12904-021-00879-z)
Supplement: Supplementary file 1 — Additional file 1: Supplementary Figure 1. Trend of Terminally Ill Non-Cancer Patients With/Without PCCS from 2011 to 2019. Supplementary Figure 2. Trend of Terminally Ill Non-Cancer Patients Receiving PCCS, Palliative Home Care and Palliative Inpatient Care from 2011 to 2019. [file 12904_2021_879_MOESM1_ESM.docx]

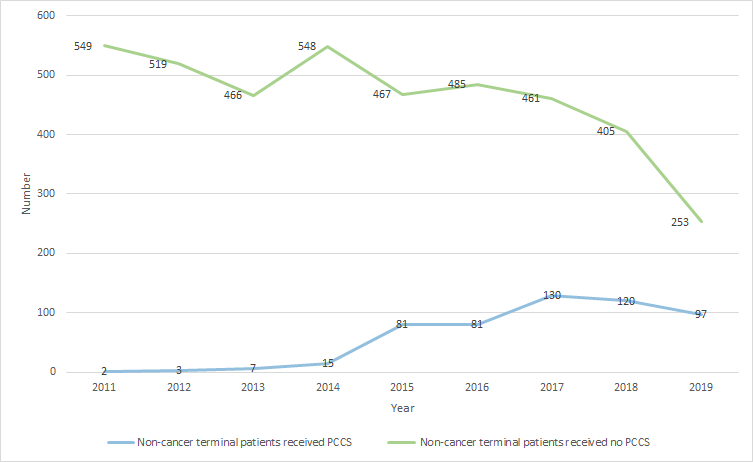


**Supplementary Figure 1.** Trend of Terminally Ill Non-Cancer Patients With/Without PCCS from 2011 to 2019


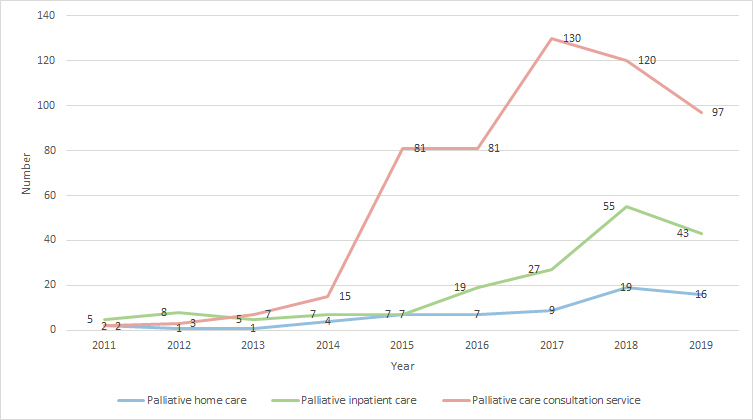


**Supplementary Figure 2.** Trend of Terminally Ill Non-Cancer Patients Receiving PCCS, Palliative Home Care and Palliative Inpatient Care from 2011 to 2019
